# Editorial to “Improvement in respiratory function and exercise tolerance following video‐assisted thoracoscopic diaphragm plication for symptomatic iatrogenic persistent diaphragm paralysis after radiofrequency catheter ablation”—An essential respiratory physiology every electrophysiologist should know‐

**DOI:** 10.1002/joa3.13064

**Published:** 2024-05-14

**Authors:** Tatsuya Hayashi, Hideo Fujita

**Affiliations:** ^1^ Division of Cardiovascular Medicine Saitama Medical Center, Jichi Medical University Saitama Japan

**Keywords:** atrial fibrillation, diaphragm plication, phrenic nerve pasly

Editorial comment on “Improvement in respiratory function and exercise tolerance following video‐assisted thoracoscopic diaphragm plication for symptomatic iatrogenic persistent diaphragm paralysis after radiofrequency catheter ablation.^1^”

Complications of catheter ablation for atrial fibrillation include right phrenic nerve palsy. In conventional radiofrequency (RF) ablation, this complication is known to occur during procedures such as superior vena cava (SVC) isolation or right pulmonary vein isolation. While improvements have been observed with treatment modalities such as high‐power short‐duration ablation,^2^ complete prevention of right phrenic nerve palsy remains challenging. Catheter ablation using cryoballoon, introduced after RF ablation, is considered a safer treatment option for atrial fibrillation. However, it is essential to note that compared to RF ablation, cryoballoon ablation has been associated with a higher incidence of right phrenic nerve palsy at the time of discharge after catheter ablation.^3^ Recent evidence has shown that in cases of persistent atrial fibrillation treated with cryoballoon ablation, there is a higher incidence of phrenic nerve palsy, particularly in long‐standing persistent atrial fibrillation cases.^4^ As ablation procedures for persistent atrial fibrillation continue to be explored and utilized more frequently, the likelihood of encountering this complication may increase. Phrenic nerve palsy is often asymptomatic and may spontaneously resolve in many cases, leading it to be perceived as a relatively benign complication. However, some patients may experience severe symptoms, warranting careful attention.

In this report by Kasai et al., a case of respiratory failure resulting from right phrenic nerve palsy following catheter ablation for atrial fibrillation is described.^1^ While phrenic nerve palsy often does not cause symptoms because of adequate oxygenation by the unaffected lung, the patient in this case, who was elderly and obese, exhibited significant symptoms after the onset of right phrenic nerve palsy. The mechanism of respiratory distress because of phrenic nerve palsy involves “paradoxical breathing” during lung expansion, wherein the flaccid diaphragm on the affected side is drawn toward the pulmonary hilum by negative pressure from the unaffected lung, reducing the inspiratory volume of the unaffected lung.

Given that phrenic nerve palsy often resolves over time, observation may suffice as a treatment strategy, even in cases where symptoms are present. However, this report suggests that more aggressive intervention may be warranted in cases of severe symptoms. One such intervention involves surgical plication of the affected diaphragm to reduce its flexibility, thereby inhibiting the “rebound” of air from the affected lung to the unaffected lung during lung expansion. This methodically simple surgical procedure can be performed relatively noninvasively using thoracoscopic techniques. In the reported case, dyspnea improved immediately after surgery, and significant improvement was observed in respiratory function tests and X‐ray images. This case highlights the importance of timely and appropriate intervention in managing phrenic nerve palsy following catheter ablation for atrial fibrillation. In another report, a patient who presented with right phrenic nerve palsy after cryoballoon ablation and was similarly treated with robot‐assisted thoracoscopic surgery was also found to be highly obese.^5^ It is important to note that such obese patients are at high risk of developing respiratory distress because of phrenic nerve palsy. The occurrence of phrenic nerve palsy following catheter ablation for atrial fibrillation is not uncommon, and it is essential to be aware that symptoms may occasionally deteriorate significantly.

Healthcare professionals performing catheter ablation for atrial fibrillation need to have knowledge not only of cardiac electrophysiology but also of respiratory physiology. Moreover, they should be aware of the existence of effective surgical treatments like this.

Another crucial point we must remember is that in phrenic nerve palsy, prevention is paramount over treatment. It is imperative that we prioritize preventive measures to mitigate the risk. Even with high‐power short‐duration ablation, there remains a risk of phrenic nerve palsy. Therefore, to ensure effective phrenic nerve pacing, SVC isolation under general anesthesia with muscle relaxants should be avoided unless reversal agents are administered.

## CONFLICT OF INTEREST STATEMENT

Authors declare no conflict of interests for this article.

## ETHICS APPROVAL STATEMENT

Yes.

## STATEMENTS RELATING TO OUR ETHICS AND INTEGRITY POLICIES

N/A.

## PATIENT CONSENT STATEMENT

Yes.

## CLINICAL TRIAL REGISTRATION

N/A.

## PERMISSION TO REPRODUCE MATERIAL FROM OTHER SOURCES

Yes.
